# An Uncommon Meridional Globe Rupture due to Blunt Eye Trauma

**DOI:** 10.1155/2018/1808509

**Published:** 2018-09-18

**Authors:** Krishan Kumar, Rick Figurasin, Swati Kumar, Muhammad Waseem

**Affiliations:** ^1^Department of Pediatrics, Nassau University Medical Center, 2201 Hempstead Turnpike, East Meadow, NY 11554, USA; ^2^Transitional Rotating Intern, Department of Emergency Medicine, Nassau University Medical Center, 2201 Hempstead Turnpike, East Meadow, NY 11554, USA; ^3^SUNY Downstate Medical Center, 450 Clarkson Avenue, Brooklyn, NY 11203, USA; ^4^Lincoln Medical & Mental Health Center, 234 East 149^th^ Street, Bronx, New York, USA

## Abstract

Open globe injury (OGI) is a severe form of eye trauma. It is an important cause of monocular blindness worldwide. Ruptures from blunt trauma are most common at the sites where the sclera is thinnest, at the insertions of the extraocular muscles, and at the limbus. Most often, rupture is equatorial. We present a unique case of open globe injury due to blunt ocular trauma from a thrown rock that resulted in a meridional rupture of the eye. The pertinent literature is reviewed.

## 1. Introduction

Ocular trauma has resulted in uniocular blindness in around 18 million patients globally with a greater incidence in children [1]. Literature has suggested that around 3.3-5.7 million children under 15 years of age suffer from ocular trauma every year in the world with pediatric ocular trauma being more prevalent in males [1]. Pediatric globe injury has been suggested to occur at a rate of 2-3.8/100,000 in the United States [2]. Open globe injury is injury to the cornea or sclera. It is usually the result of penetrating eye trauma and thus are not so common [3, 4]. In a review of traumatic pediatric open globe injuries in an urban U.S. population, blunt rupture was found to be the cause, 34% of the time. This is in contrast to penetrating trauma which accounts for 54% of the incidence of open globe injury in children [5]. We present a unique case of open globe injury due to blunt ocular trauma from a thrown rock that resulted in a meridional rupture of the eye. The Birmingham Eye Trauma Terminology System (BETTS) was utilized to classify the traumatic eye injury as an open globe injury [6].

## 2. Case Presentation

A previously healthy 14 year-old boy presented to the Emergency Department (ED) for evaluation of right eye injury that was sustained one to two hours prior to arrival. According to the patient, he was standing on a street corner on his way to school when he was struck in the right eye with a rock thrown from a passing car. He reported no loss of consciousness and no other injury. He denied nausea or vomiting. His past medical history and family history were not significant. The patient did not have any prior ocular injuries, history of ocular disease, or prior ocular surgeries. The patient reported that he does not wear glasses.

**Figure 1:**
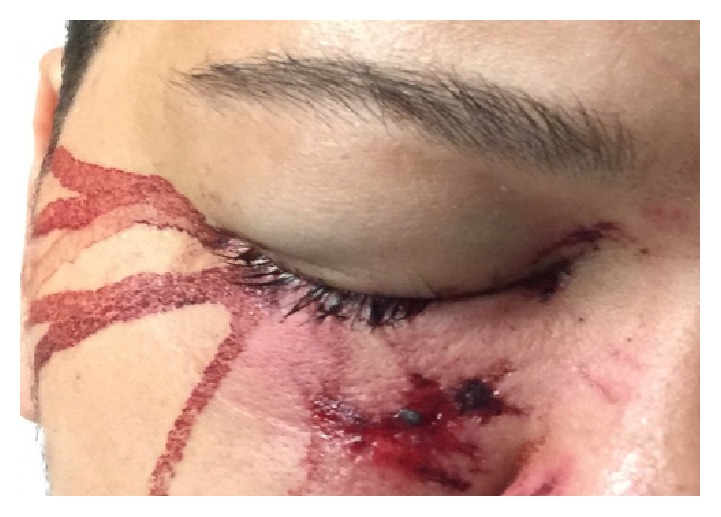
Patient presentation after reported right eye injury from a thrown rock.

In the ED, he was alert, awake, answering questions, and following commands with a GCS of 15. His only complaint was pain to the right eye. His vital signs were as follows: temperature 98°F, heart rate 79 beats/minute, respiratory rate 18 breaths/minute, blood pressure 151/73 mmHg, and oxygen saturation 99% on room air. On examination, blood was oozing and pooling in his right eye with eye lid swelling and surrounding abrasions (Figure 1).

The patient expressed severe photophobia in his right eye and was unable to open it. Detailed examination of the right eye could not be performed due to the possibility of right ocular globe rupture. A metal eye shield was placed on the patient's right eye for protection. His left eye refractory status was normal with a visual acuity of 20/20. The patient had a negative battle sign, no hemotympanum, no rhinorrhea, and no cervical spine tenderness. The remainder of his examination was noncontributory.

**Figure 2:**
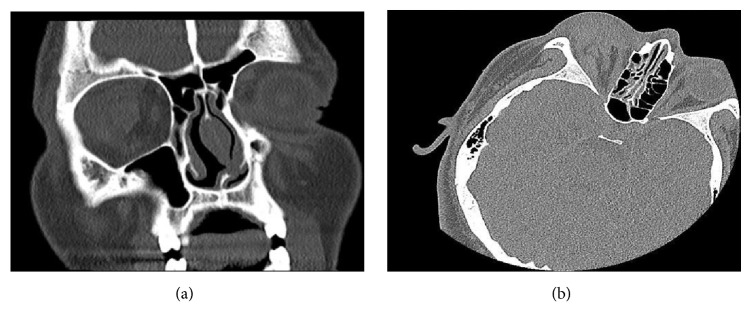
Right globe rupture on nonenhanced CT scan of the orbits.

A non-enhanced CT scan of the orbits was obtained and revealed rupture of the right globe with associated orbital and periorbital swelling with intraconal hematoma (Figure 2).

An ophthalmology consultation was obtained. He was then taken to the operating room where a 20 mm linear laceration was noted crossing the iris and sclera with uvea protruding. The laceration was repaired. The patient tolerated the procedure well and was followed-up in the clinic on the next day. The patient received an explanation regarding the poor prognosis of his right eye vision. The open globe injury caused blindness in his right eye. The patient was advised to wear protective eye gear to prevent future eye injuries while participating in activities that carry a risk of eye injury.

## 3. Discussion

The paucity of globe rupture blunt eye trauma is likely due to the force required to cause such an injury. The eye is a fluid-filled structure that is relatively not compressible. An object moving at high speed transfers kinetic energy to the globe resulting in compression of the structures of the eye. This raises the intraocular pressure, which creates shear forces at the interface of tissues with different elasticities. The resulting high stresses eventually cause tissue tearing, which result in decompression through a ruptured hole in the eye [3, 7]. The amount of blunt force required to induce open globe injury is extremely high. The normal intraocular pressure of the eye is between 12-22 mmHg. It is reported that, during rupture, pressure needs to exceed 6800 mmHg (approximately 132 PSI). The average rupture pressure of the eye occurred at 0.91 +/- 0.29 MPa or Mega pascals (6826 mmHg, 132 PSI). Surprisingly, the average pressure at which there was a 50% risk of rupture was 0.90 MPa (6750 mmHg, 131 PSI). This leaves only a 0.01 MPa or 1 PSI difference from 100% rupture [8]. Another study reported a mean rupture pressure of 0.97 MPa (7275.60 mmHg, 140.6 PSI) [5]. Through computational simulations, globe rupture was predicted to occur at pressures exceeding 17.21 MPa at the corneo-scleral interface and 1.01 MPa at the vitreous chamber [9]. These pressures roughly translate to 129,085.59 mmHg (2496 Psi) and 7575.62 mmHg (146 Psi), respectively. Interestingly enough, the investigators in these studies noted that the primary site of rupture occurred at the equatorial region of the eye [10]. Studies by Bisplinghoff et al. echoed similar findings when 16 of their ruptured eyes occurred at the equator versus 4 in the meridional direction [7]. In our case, the meridional region was found to be the primary site of rupture in contrast to more commonly reported, equatorial region ruptures. Thus our patient highlights a unique presentation of open globe injury caused by blunt trauma resulting in meridional rupture of the eye.

Blunt trauma to the eye results in various injuries to the ocular structures, which range from subconjunctival hemorrhages to corneal abrasions. One of the more common serious presentations after blunt eye trauma is the presence of a hyphema due to a tear of the ciliary body or iris, resulting in the anterior chamber being filled with blood [10–13]. Secondary hemorrhage or subsequent bleeding from the traumatic hemorrhage may occur within a week or result in a poor outcome [14]. Secondary glaucoma may also result from a hyphema. Thus it is imperative to adequately evaluate and manage blunt ocular trauma and identify not only equatorial ocular rupture, but meridional as well. Another common complication of blunt ocular trauma is abrasion of the cornea. With corneal abrasion one can witness photophobia, eye pain, and discomfort, along with foreign body sensation. Corneal abrasions can lead to iritis, bacterial keratitis, and ulcers in the cornea [15]. Injury to the sclera and cornea can also lead to endophthalmitis, cataracts, and retinal detachment [2]. Thus with the above complications, it is imperative to evaluate for blunt ocular trauma that has resulted in both equatorial and meridional rupture of the eye.
